# ﻿Four new inquiline social parasite species in the dolichoderine ant genus *Tapinoma* (Hymenoptera, Formicidae)

**DOI:** 10.3897/zookeys.1202.120478

**Published:** 2024-05-15

**Authors:** Stefan P. Cover, Christian Rabeling

**Affiliations:** 1 Museum of Comparative Zoology, Harvard University, 26 Oxford Street, Cambridge, MA 02138, USA Harvard University Cambridge United States of America; 2 Department of Integrative Taxonomy of Insects, Institute of Biology, University of Hohenheim, Garbenstraße 30, 70599 Stuttgart, Germany University of Hohenheim Stuttgart Germany; 3 KomBioTa – Center for Biodiversity and Integrative Taxonomy Research, University of Hohenheim & State Museum of Natural History Stuttgart, Stuttgart, Germany University of Hohenheim & State Museum of Natural History Stuttgart Stuttgart Germany; 4 Social Insect Research Group, School of Life Sciences, Arizona State University, 550 E Orange Street, Tempe, AZ 85281, USA Arizona State University Tempe United States of America

**Keywords:** Brood parasitism, Dolichoderinae, Formicidae, integrative taxonomy, inquilinism, social parasitism

## Abstract

Four new inquiline social parasites are described in the dolichoderine ant genus *Tapinoma* from the Nearctic region, and keys are provided for queens and males of the Nearctic *Tapinoma* species. The new social parasite species represent the first inquiline species in the genus *Tapinoma* and the first confirmed inquilines known from the ant subfamily Dolichoderinae. The four new species appear to be workerless inquilines that exploit a single host, *Tapinomasessile* (Say), and they represent at least two distinct life history syndromes. *Tapinomaincognitum* Cover & Rabeling, **sp. nov**. is highly derived morphologically and is a host-queen-tolerant inquiline. In contrast, *T.inflatiscapus* Cover & Rabeling, **sp. nov.** shows a lesser degree of morphological modification and appears to be a host-queen-intolerant social parasite. The life history of *T.pulchellum* Cover & Rabeling, **sp. nov.** is presently unknown, but its close similarity to *T.incognitum* suggests that it is also a host-queen-tolerant inquiline. The life history of *T.shattucki* Cover & Rabeling, **sp. nov.** is still uncertain. Our findings provide novel insights into the complex biology of ant inquiline life history syndromes.

## ﻿Introduction

Social parasitism, the dependence of one social insect species on another during colony founding and/or during its complete life cycle, is one of the most intriguing life history phenomena found in the eusocial Hymenoptera (see Hölldobler and Wilson 1990; Bourke and Franks 1991; Buschinger 2009; Rabeling 2021 for general reviews). Most ant social parasites belong to one of three basic life history syndromes. Temporary social parasites require the assistance of a host species during colony founding only, and established colonies of the parasite produce numerous workers and are fully independent (Wheeler 1904; Borowiec et al. 2021). Dulotic social parasites establish new colonies as temporary social parasites, but in mature colonies the parasite workers raid host nests and steal their brood, some of which is eaten. The remaining brood complete development to become host workers that feed and maintain the parasite colony (Hölldobler and Wilson 1990). The vast majority of dulotic parasites are completely dependent on their host workers.

The third major life history syndrome of ant social parasites is permanent inquilinism (Wilson 1971). Inquiline ants live within the nests of their hosts for their entire life cycle, excepting a brief period during which dispersal (and mating in some species) occurs. In most cases the inquiline queen coexists with one or more host queens but “castrates” the colony by suppressing or greatly reducing the production of host sexual forms and substituting the production of parasite sexual offspring (Kutter 1968; Rabeling and Bacci 2010). In others, the inquiline somehow eliminates the host queen(s) or is adopted successfully only in host colonies that have lost their queen (Wilson 1971; Rabeling et al. 2019). The inquiline worker caste is usually absent or produced in very small numbers. Inquilines seem to be extremely rare, and their populations appear to be few in number, small in size, and sporadically distributed (Wilson 1963). In addition, inquiline ants are usually small and inconspicuous relative to their hosts and are thus seldom noticed or collected, reinforcing the impression of rarity. As a result, our knowledge of the ecology, life history, and distribution of most inquiline ant species is fragmentary.

Inquilinism has evolved at least 57 times convergently across the formicoid clade of the ant tree of life, and 88 inquiline species have been confirmed to date (Gray and Rabeling 2023). The majority of inquiline species are concentrated in the ant subfamilies Myrmicinae and Formicinae. Curiously, true inquilines are unknown from the subfamily Dolichoderinae, though temporary social parasites occur with some frequency. Temporary social parasites have been reported from the dolichoderine genera *Arnoldius*, *Azteca*, *Bothriomyrmex*, *Chronoxenus*, and *Dorymyrmex* (Trager 1988; Dubovikoff 2005; Longino 2007; Guerrero et al. 2010). Based on the morphology of the queens, inquilinism has been suggested for *Aztecananogyna* and *Aztecadiabolica* (Longino 2007; Guerrero et al. 2010). Both are known from only a handful of specimens collected by canopy fogging or in flight-interception traps (Longino 2007; Guerrero et al. 2010), and their hosts and life-histories remain unknown.

Until the present, social parasites of any kind were unknown in the dolichoderine genus *Tapinoma.* Currently, 72 extant species are recognized in the genus *Tapinoma* and the genus is distributed globally (Bolton 2024). The North American ant fauna contains only four described species (Fisher and Cover 2007). *Tapinomalitorale* Wheeler, *Tapinomaschreiberi* Hamm, and *Tapinomasessile* (Say) are native, whereas *Tapinomamelanocephalum* (Fabricius) is a global tramp species established in Florida (Hamm 2010; Deyrup 2017). Here, we report the first inquilines discovered in the genus *Tapinoma*. Curiously, the new *Tapinoma* inquilines all parasitize a single host species, *Tapinomasessile*. The evolution of several inquilines against the complex population genetic backdrop of a single, widespread host species makes this host-parasite complex unique in the context of ant social parasitism and deserving of further examination.

## ﻿Materials and methods

### ﻿Specimens examined

In addition to new collections, specimens from the insect collections listed below were examined for this study:

**CASC** California Academy of Sciences, San Francisco, CA, U.S.A.

**CRC** C. Rabeling Collection, University of Hohenheim, Stuttgart, Germany

**LACM** Los Angeles County Museum of Natural History, Los Angeles, CA, U.S.A.

**MCZC** Museum of Comparative Zoology, Harvard University, Cambridge, MA, U.S.A.

**UCDC** Bohart Museum of Entomology Collection, University of California at Davis, CA, U.S.A.

### ﻿Morphometric measurements

Specimens were examined and measured using a Leica MS5 stereomicroscope fitted with a stage micrometer. Measurements were taken at 25× magnification. Morphometric conventions and indices follow Bolton (1987) and modifications described in Rabeling et al. (2007). Morphometric measurements and indices are defined as follows in Table 1.

**Table 1.** Morphometric measurements and indices.

HL	Head Length	Length of the head in full face view, excluding mandibles, measured in a straight line from the midpoint of the anterior clypeal margin to the midpoint of the posterior margin of the head. In species where the posterior margin or the clypeal margin (or both) is concave, the measurement is taken from the midpoint of a transverse line spanning the anteriormost or posteriormost projecting points, respectively.
HW	Head Width	Maximum width of head, not including the eye.
CI	Cephalic Index	HW*100/HL
SL	Scape Length	Maximum straight-line length of the antennal scape excluding the basal constriction or neck close to the condylar bulb.
SI	Scape Index	SL*100/HW
ML	Mesosoma Length	Diagonal length of the mesosoma in profile from the point at which the pronotum meets the cervical shield to the posterior base of the metapleuron.

## ﻿Results

### ﻿Key to the queens of *Tapinoma* species occurring in the Nearctic region north of Mexico

**Table:** 

1	Maxillary palp segments 2–4 strikingly flattened and notably broader than palp segments 1, 5, and 6. Labial palp segments 2 and 3 also flattened. Workers distinctly bi-colored with dark, almost black head and mesosoma contrasting sharply with pale yellow-white metasoma and appendages. [pantropical tramp, heated greenhouses, and buildings, outdoors in southern Florida, exotic]	***T.melanocephalum* (Fabricius)**
–	Maxillary and labial palp segments subcylindrical, not strikingly flattened and broad. Workers (if present) often uniformly colored; if bicolored, different from pattern described above	**2**
2	At least some erect hairs present on gastric tergites 1–3, sometimes short erect hairs present on mesosomal dorsum as well. Pubescence on antennal scapes short but suberect	**3**
–	Erect hairs absent on mesosomal dorsum and gastric tergites 1–3. Pubescence on antennal scapes variable but generally appressed	**4**
3	Short erect hairs present on mesosomal dorsum and gastric tergites 1–3. Antennal scapes long, projecting well beyond the posterior corners of the head, and reaching maximum diameter between the antennal insertion and the mid-point. Short but dense suberect pubescence present on scapes and most dorsal body surfaces. Palp formula (5,4). (Fig. 2)	***T.inflatiscapus* sp. nov.**
–	Erect hairs absent on mesosomal dorsum. Antennal scapes short, barely, or just reaching the posterior corners of the head, subcylindrical, tapering in diameter only near the antennal insertion and near the apex. Palp formula (6,4). [subtropical Florida, the Caribbean region, arboreal]	***T.litorale* Wheeler**
4	In full-face view, head trapezoidal, broadest near the posterior border well behind the large compound eyes, sides straight or slightly convex (except for the broadly rounded posterior corners), and strongly converging anteriorly. In lateral view, dorsal petiolar scale absent. In dorsal view, anterodorsal margin of petiole convex; petiole broadest at midpoint	**5**
–	In full-face view, head shape variable, either subrectangular or sides of head convex with head widest at level of eyes. In lateral view, petiolar scale either absent or forming a small, rounded node. When small node present, shape of petiole when viewed from the rear trapezoidal or subrectangular, dorsal margin slightly convex to flat or concave. If petiolar scale absent, head widest at level of eyes in full-face view	**6**
5	Queen bicolored with head and mesosoma orange to dull red, often with patches of darker infuscation, forming a strong contrast with the uniformly black or brown gaster. Note: workers combining the following: head and mesosoma orange to red, metasoma brown to black; in full-face view, posterior margin of head concave. [mountains of southern Nevada, and central to southern California]	***T.schreiberi* Hamm**
–	Queen usually uniform gray or brownish gray, variable in size. Note: workers uniform gray to yellowish brown, sometimes weakly bicolored, but never as described above; in full-face view, posterior margin of head usually straight or weakly convex, rarely slightly concave. [omnipresent from sub-boreal Canada south through the Mexican highlands]. (Fig. 5)	***T.sessile* (Say)**
6	In lateral view, petiolar scale absent. In lateral view, propodeum forming a single, flat declivitous surface. Palp formula (5,4). (Fig. 4)	***T.shattucki* sp. nov.**
–	In lateral view, petiole with a small dorsal rounded node. In lateral view, propodeum with dorsal and posterior surfaces that meet to form a convexity. Palp formula either (4,3) or (5,4)	**7**
7	Palp formula (5,4). Shape of petiole when viewed from the rear subrectangular, dorsal margin slightly convex to flat. In side view, propodeum subangulate with dorsal face notably shorter than posterior face; posterior face slightly concave. Anterior clypeal margin flat. (Fig. 3)	***T.pulchellum* sp. nov.**
–	Palp formula (4,3). Shape of petiole when viewed from the rear trapezoidal, broadest at apex, dorsal margin concave, rarely flat. In side view, propodeum broadly rounded, forming an even convexity. Anterior clypeal margin with small median impression. (Fig. 1)	***T.incognitum* sp. nov.**

### ﻿Key to the males of *Tapinoma* species occurring in the Nearctic

**Table:** 

1	Males minute, wings short, deformed, clearly non-functional. Number of antennal segments reduced to 12	**2**
–	Males variable in size, fully alate. With 13 antennal segments	**3**
2	Anterior border of clypeus weakly emarginate in full-face view. Cutting edge of mandible with 2–4 small denticles (clearly visible at high magnification) in addition to apical tooth. (Fig. 1)	***T.incognitum* sp. nov.**
–	Anterior border of clypeus flat in full-face view. Cutting edge of mandible smooth, lacking distinct subapical teeth or denticles; apical tooth present. (Fig. 3)	***T.pulchellum* sp. nov.**
3	Males minute (< 2 mm), fragile, gnat-like, pale yellow or pale gray	**4**
–	Males larger (generally > 2.5 mm), more robust, solid gray or black	**5**
4	Antennal scapes just reaching or barely surpassing the posterior border of the head. Maxillary palps delicate, filiform, segments uniformly thin and cylindrical	***T.litorale* Wheeler**
–	Antennal scapes long, surpassing the posterior border of the head by 2–3× the maximum diameter of the scape. Maxillary palps robust, segments 3 and 4 thicker than the preceding and terminal two segments (seldom collected)	***T.melanocephalum* (Fabricius)**
5	Very short, fine erect hairs present on mesosomal dorsum and gastric tergites. Pubescence on head and mesosoma short, dense, and suberect. (Fig. 2)	***T.inflatiscapus* sp. nov.**
–	Erect hairs absent on mesosoma and first three gastric tergites. Pubescence on head, and mesosoma variable, but appressed on most body surfaces	**6**
6	In full-face view, sides of head strongly convex, head nearly circular exclusive of the compound eyes. As small or smaller than host workers. (Fig. 4)	***T.shattucki* sp. nov.**
–	In full-face view, head variable in shape with sides of head converging anteriorly; head shape but not as described above. Body size variable as well; often as large or larger than nestmate workers	**7**
7	In full-face view, head distinctly longer than broad. Body color dark brown to black; tips of legs and antennal flagellum reddish-yellow	***T.schreiberi* Hamm**
–	In full-face view, head often as broad or broader than long. Body color brownish, yellowish, or battleship gray. (Fig. 5)	***T.sessile* (Say)**

### ﻿Species accounts

#### ﻿ Tapinoma incognitum

Taxon classificationAnimaliaHymenopteraFormicidae

Cover & Rabeling sp. nov.

952694EE-D45D-5470-9120-A8ED6E68BB88

https://zoobank.org/6E385B56-D69F-4EB7-A75C-88C2D7D23A67

##### Diagnosis.

**Figure 1.**
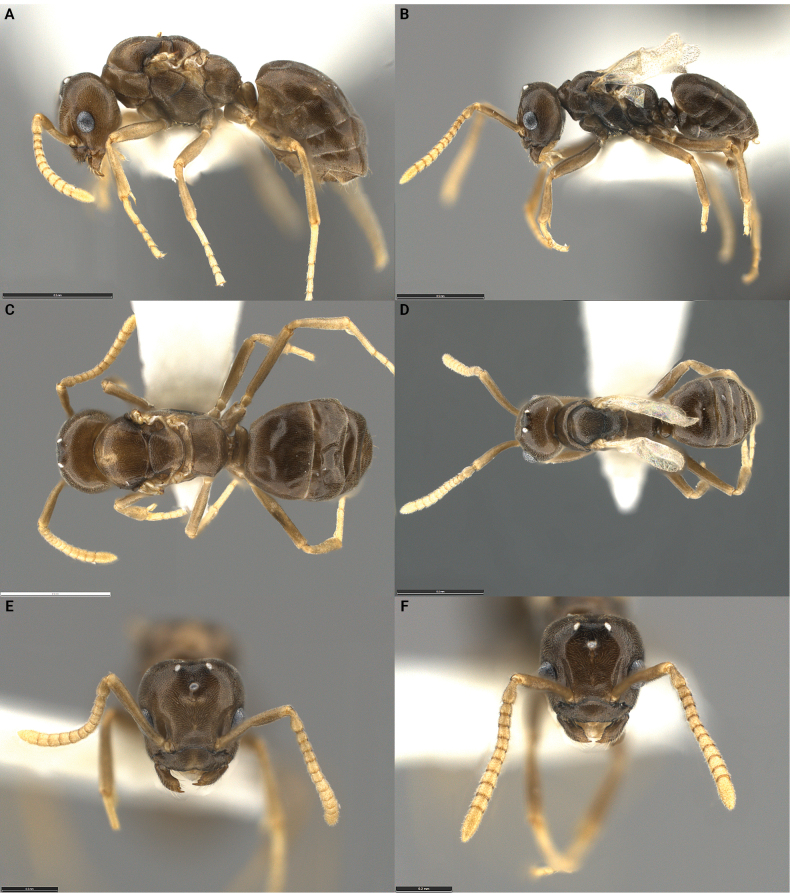
Morphological comparison of the *Tapinomaincognitum* holotype queen **A, C, E** and a paratype male **B, D, F** in lateral **A, B** dorsal **C, D** and full-face **E, F** view. The type series was collected in Alumbed Hollow in Utah and belongs to a single nest series with the collection code SPC 7749. Scale bars: 0.5 mm (**A–D**); 0.2 mm (**E, F**).

A workerless, host-queen-tolerant inquiline social parasite of *Tapinomasessile* showing morphological and life history traits of the inquiline syndrome. Both females and males are miniaturized (i.e., smaller than the host workers), alate, and morphologically complete (Fig. 1, Table 2). Females eclose with intact wings, but the wings are fragile and quickly deciduous. Males are brachypterous. Females have a reduced 4,3 palp formula, anterior clypeal border with weak median concavity, denticulate mandibles with only 2–4 denticles. In side view petiole with low, rounded node; viewed from the rear dorsal margin concave (rarely flat). Males similar in size and overall habitus to females but often darker in color and easily recognized by their extruding genitalia. Males have a reduced 5,3 palp formula and only 12 antennal segments. Females of *T.incognitum* are closely similar in habitus to those of *T.pulchellum* sp. nov., but can be easily distinguished by palp count, concave anterior clypeal border, mandibular dentition, and propodeal profile.

**Table 2.** Morphological and life history traits characteristic of the inquiline syndrome in *Tapinoma* ants. Morphological reductions are determined by comparisons to the host, *Tapinomasessile*, which is included in this table (traits modified from Kutter 1968; Wilson 1971, 1984; Rabeling et al. 2019; Prebus et al. 2023).

	Host	Social parasites			
	*T.sessile*	*T.shattucki*	*T.inflatiscapus*	*T.incognitum*	*T.pulchellum*
Worker caste absent	–	+	+	+	? (+)
Multiple egg laying host queens present (host polygyny)	+	? (–)	–	+	?
Multiple egg laying parasite queens present in host colony (parasite polygyny)	n/a	+	?	+	?
Parasite queen coexists with host queen (host queen tolerance)	n/a	? (–)	–	+	? (+)
Adelphogamy (inside nest mating)	–	? (–)	?	+	?
Gynaecomorphism (gyne-like male morphology)	–	–	–	+	+
Fragmented populations, limited geographic distribution	–	+	+	+	+
	(North America)	(2 localities in MA)	(UT, CO)	(type locality, UT)	(type locality, NC)
Reduced body size	–	+	+	+	+
		(size of host worker)	(size of host worker)	(smaller than host worker)	(smaller than host worker)
Exoskeleton becomes thinner and less pigmented	–	+	+	+	+
Number of antennal segments reduced in females	–	–	–	–	–
	(♀: 12)	(♀: 12)	(♀: 12)	(♀: 12)	(♀: 12)
Number of antennal segments reduced in males	–	–	–	+	+
	(♂: 13)	(♂: 13)	(♂: 13)	(♂: 12)	(♂: 12)
Number of maxillary and labial pals (palp formula) reduced in females	–	+	+	+	+
	(♀: 6,4)	(♀: 5,4)	(♀: 5,4)	(♀: 4,3)	(♀: 5,4)
Number of maxillary and labial pals (palp formula) reduced in males	–	+	–	+	+
	(♂: 6,4)	(♂: 5,4)	(♂: 6,4)	(♂: 5,3)	(♂: 5,4)
Reduced mandibular dentition	–	–	–	+	+
	14 teeth	(10–11 denticles)	(11 denticles)	(2–4 denticles, plus apical tooth)	(only apical tooth)
Reduced wings in females	–	–	–	+	–
	(♀ capable of flying)	(♀ capable of flying)	(♀ capable of flying)	(♀: wings deciduous)	(♀: winged)
Reduced wings in males	–	–	–	+	+
	(♂ capable of flying)	(♂ capable of flying)	(♂ capable of flying)	(♂: brachypterous)	(♂: brachypterous)
Petiole thickened	–	–	–	+	+

##### Description.

***Holotype*** queen: HL 0.53, HW 0.53, SL 0.44, ML 0.82, CI 100, SI 83. Head in full-face view nearly square, dorsal margin straight with corners evenly rounded. Anterior margin of clypeus with shallow median impression; posterior border rounded, not projecting forward between antennal insertions. Mandibles reduced, barely touching each other when mandibles are fully closed; apical tooth well developed, cutting edge of mandible with 2–4 small denticles. Antennae with 12 segments, scapes relatively short, surpassing the dorsal margin of head by less than their own maximum width. Palp count 4,3. Mesosoma with typical modifications related to wing bearing. In lateral view, propodeum forming an evenly rounded convexity and lacking distinct dorsal and posterior surfaces. Orifices of propodeal spiracles slightly elevated and conspicuous. Metapleural gland orifice significantly reduced. Petiole in side view with low, blunt node; viewed from the rear trapezoidal with concave dorsal margin. Petiolar spiracles located on top of laterally extended tubercles. In dorsal view, four gastric tergites visible. Integument thin, specimens can shrivel when dried. Body surface covered with short, appressed pubescence; posterior margin of all gastric sternites and fourth gastric tergite with sparse, long setae. Color pale brown to yellowish brown, appendages pale yellow. Paratype queens (*n* = 8): HL 0.50–0.53, HW 0.50–0.53, SL 0.41–0.47, ML 0.76–0.88, CI 94–100, SI 78–94.

***Paratype*** male: HL 0.50, HW 0.47, SL 0.47, ML 0.76, CI 94, SI 100. Males small, approximately the same size as the queen, brachypterous, closely similar to the conspecific queen in habitus. Head in full-face view almost square. Eyes small, maximum diameter ~ ¼ of head length; individual ommatidia partly fused, lacking the distinct convex surface of each ommatidium; compound eyes appear to be coated with a translucent resin. Ocelli slightly elevated above the surface the head. Anterior clypeal margin with a broad, median, shallow impression. Mandibles reduced, with a single large apical tooth; denticles on cutting edge of mandible indistinct. Antennae with 12 segments, scapes shorter than the head (CI), surpassing the dorsal margin of head by twice their maximum width. Palp count 5,3. Mesosoma enlarged with typical modifications related to wing bearing. In lateral view, propodeum rounded, convex, with dorsal and posterior of approximately equal length. Metapleural gland orifice absent/reduced. Petiole small, overhung by first gastric tergite, not entirely visible in dorsal view. In dorsal view, five gastric tergites visible. Hind wings reduced to wing remnants lacking venation. Body surface covered with short, appressed pubescence, except for the antennal scapes and flagellum, which are covered by a dense, short, suberect pubescence. Color medium brown to black. Paratype males (*n* = 3): HL 0.50–0.56, HW 0.47–0.53, SL 0.44–0.47, ML 0.76, CI 94–100, SI 83–100.

##### Etymology.

When first seen in the field, the collector’s initial impression was of a *Tapinomasessile* colony infested by tiny diapriid wasps of some kind. A second look made it clear they were, in fact, tiny inquilinous ants. Hence, the species name is the nominative neuter of the Latin adjective *incognitus*, meaning unknown, unrecognized, in disguise.

##### Type locality.

U.S.A., Utah, Sevier County, Alumbed Hollow, 8.4 miles west of I-70 (Exit 71) on Salina County Frontage Rd., a dirt road paralleling I-70. GPS: 38.910°N, 111.697°W; elevation 5980’ (1823 m). Small canyon running southwest to northeast with dense, heavily grazed Gambel Oak (*Quercusgambelii*) thickets to 25’ (8 m) tall on east-facing slope. Collected by SPC (SPC 7749), 16 July 2008. Collection Notes: SPC 7749. Site heavily grazed. Dense Gambel Oak thicket; forest floor protected from grazing by oak stem density. Superficial nest under rock in pale shade of dense Gambel Oak thicket. 2-cm thick oak litter present. Humusy sand soil. Very dry conditions. ~ 500 ants, multiple host queens present. Brood was mostly eggs and young larvae; just a few parasite and host worker pupae present.

##### Type material.

***Holotype*** queen (SPC 7749, MCZENT 00806456). ***Paratype*** male (SPC 7749, MCZENT 00806457), and the following additional paratypes: 13 queens, 17 males [16-VII-2008, SPC 7749]; 35 queens, 3 males [19-VII-2009, SPC 8077]; 19 queens, 4 males [19-VII-2013, SPC 8656]. Holotype and paratypes deposited in the MCZC. Additional paratypes deposited at CASC, CRC, LACM, and UCDC.

##### Additional material.

(i) SPC 8077. Same site description as above. Site not as heavily grazed as in 2008. Nest under dead oak branch half buried in oak litter in shade. Very dry conditions. ~ 1500 ants. Multiple host queens present. Eggs and young larvae present plus some host worker pupae. No inquiline pupae seen.

(ii) SPC 8656. Same site description as above. Not as heavily grazed as in 2008. In 9 cm diameter hard, dead oak stump in shade. Very dry conditions. ~ 1000 ants. Multiple host queens present. Eggs, larvae, and a few inquiline and host worker pupae present.

##### Discussion and biology.

*Tapinomaincognitum* is known from three collections that were made at the type locality on separate occasions. All were mixed colonies containing *T.incognitum* and its host *T.sessile.* Each colony contained multiple fertile host queens, numerous host workers, and some host worker pupae. In addition, each nest contained males and females of *T.incognitum*, and, in two collections, parasite pupae. No *T.incognitum* workers were found. In each colony, several *T.incognitum* queens were observed with enlarged metasomas, implying that multiple parasite queens were reproductively active (i.e., functional polygyny of social parasite; Table 2). A striking feature of this species is the strong convergence in size and habitus between females and males (i.e., gynaecomorphism; Table 2). Males, however, are easily recognizable by their externally visible genitalia and because they are brachypterous; the wing remnants are small, crumpled, distorted, and persistent. In addition, *T.incognitum* also displays other morphological characters typical of the inquiline syndrome (Fig. 1, Table 2). Hallmark characters include reduced body size, the reduction of antennal segments in the males, and the reduction of palp segments in both queens (palp formula 4,3) and males (palp formula 5,3). The wings of queens are extremely fragile, easily deciduous, and almost certainly non-functional, and the males cannot fly. Thus, mating must take place in or around the nest.

We kept a colony alive for a few days and made some behavioral observations. It was eye-catching that the host workers carried social parasite queens as if they were pupae and the parasites retracted their appendages against their bodies and became pupae-like when carried. The host workers also regurgitated to and groomed the social parasite queens. Host and social parasite queens encountered one another often but seemed to ignore each other. This suggests that *T.incognitum* is well integrated in the host society. Collectively these morphological and life history traits indicate that *T.incognitum* is a workerless, host-queen-tolerant inquiline social parasite of *T.sessile*.

#### ﻿ Tapinoma inflatiscapus

Taxon classificationAnimaliaHymenopteraFormicidae

Cover & Rabeling sp. nov.

0740954F-A64F-540E-B2AF-A83383CB77F6

https://zoobank.org/6DB40425-2AF1-48D3-9D3A-E1B47FBB0F76

##### Diagnosis.

**Figure 2.**
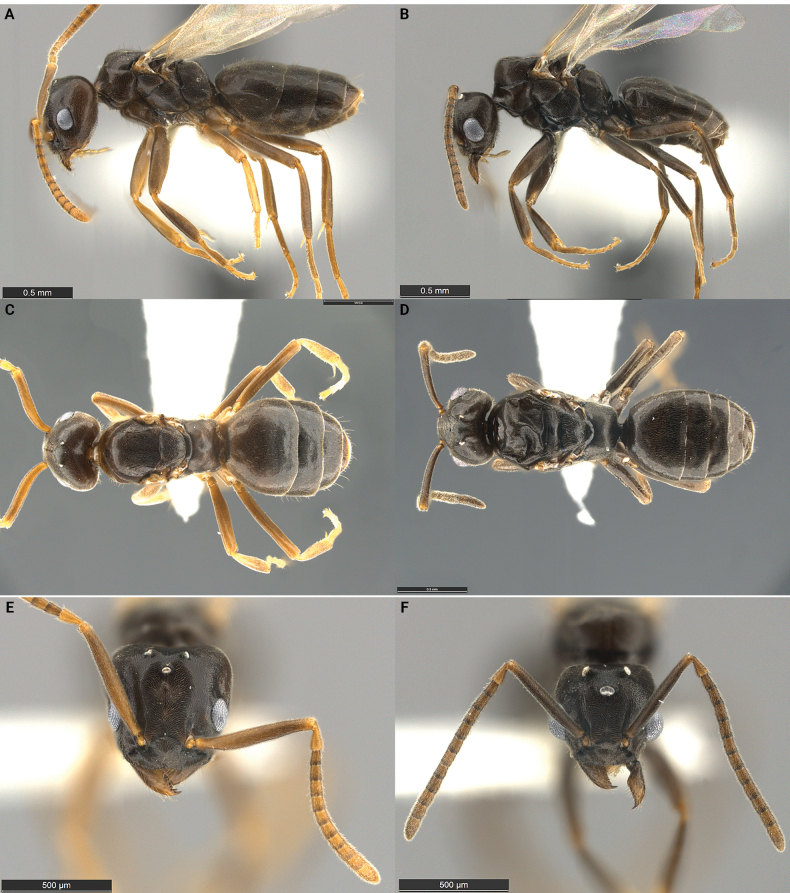
Morphological comparison of the *Tapinomainflatiscapus* holotype queen **A, E** and a paratype male **B, F** in lateral **A, B** and full-face **E, F** view. For the dorsal views of *T.inflatiscapus***C, D** dealate queen **C** and male **D** paratypes were photographed. The type series was collected at Cove Mountain in Utah and belongs to a single nest series with the collection code SPC 7816. Scale bar: 0.5 mm (**A–F**).

A unique, workerless, host-queen-intolerant inquiline social parasite of *Tapinomasessile* with relatively few morphological adaptations to its parasitic lifestyle. Inquiline females and males are equal in size, smaller than the host females and males, and approximately the size of host workers (Fig. 2, Table 2). Both females and males are winged and seem capable of flying. Females have a reduced 5,4 palp formula whereas males have the same palp formula (6,4) as host males. Both sexes of *T.inflatiscapus* are easily distinguished from those of all other North American congeners by the presence of short, erect hairs on the dorsal surface of the head, the mesosomal dorsum, and the first gastric tergite. In addition, the antennal scapes are covered by short, dense, suberect pubescence and may have one or two erect hairs near the distal end. Lastly, in females, the scape reaches its maximum diameter between the mid-point and the antennal insertion, not posterior to the mid-point as in many other *Tapinoma* species.

##### Description.

***Holotype*** queen: HL 0.76, HW 0.76, SL 0.74, ML 1.15, CI 100, SI 96. Head distinctly heart shaped, approximately as wide as long. Clypeus broad, anterior margin flat or slightly convex, median impression absent; posterior margin rounded, projecting between antennal insertions. Mandibles well developed, overlapping when closed; first three apical teeth well developed, continually decreasing in size from apex to base. Eleven teeth and denticles present, most denticles on cutting edge of mandible ill defined. Antennae with 12 segments, scapes relatively long, easily surpassing the dorsal margin of head; scape somewhat dorsoventrally flattened, basal third slightly curved. Scape widest between the insertion and the mid-point. Palp count 5,4. Mesosoma robust with typical modifications related to wing bearing. In side view, propodeum with dorsal surface approximately one third as long as posterior surface. Metapleural gland orifice large and rounded in oblique view; orifice guarded by long setae pointing inwards. Petiole reduced, overhung by first gastric tergite. In dorsal view, four gastric tergites visible. Integument thin. Body surfaces with micro-sculpture resembling a honeycomb. Entire body with dense, short, suberect to erect pubescence, including head and antennal scapes. Short erect hairs present on dorsal surface of head and mesosoma. Color pale to medium brown, legs and antennae paler, yellowish brown. Paratype queens (*n* = 8): HL 0.65–0.76, HW 0.65–0.76, SL 0.68–0.76, ML 1.03–1.15, CI 92–100, SI 96–113.

***Paratype*** male: HL 0.62, HW 0.62, SL 0.62, ML 1.12, CI 100, SI 100. Males similar in size to the females, head as wide as long (CI). Eyes large, maximum diameter ~ ⅓ of head width. Ommatidia clearly separated from each other; each ommatidium with a distinct convex surface. Ocelli slightly elevated above the surface of the head, but forming a raised, triangular turret in side view. Anterior margin of clypeus flat, median impression absent; posterior margin rounded, projecting between antennal insertions. Mandibles well developed with a single large apical tooth and ~ 18 denticles on cutting edge of mandible. Antennae with 13 segments, scapes surpassing the posterior border of the head by a bit less than ½ their length. Palp formula 6,4. Mesosoma robust with typical modifications related to wing bearing. In side view, propodeum with dorsal surface ~ ⅓ as long as the posterior surface. Metapleural gland orifice pointing backwards, circular in posterior view. Petiole visible in dorsal view. In dorsal view, six gastric tergites visible. Front and hind wings well developed. Dorsal body surfaces with short, suberect to erect pubescence, including head and antennal scapes. Short, erect hairs present on dorsal surfaces of head and mesosoma, long erect hairs irregularly dispersed over the body. Color uniformly pale to yellowish brown. Paratype males (*n* = 6): HL 0.53–0.71, HW 0.53–0.71, SL 0.59–0.65, ML 0.91–1.12, CI 90–100, SI 92–117.

##### Etymology.

In *T.inflatiscapus* females, the antennal scape reaches its maximum diameter between the mid-point and the antennal insertion instead of posterior to the mid-point as in other *Tapinoma.* This unique, diagnostic morphological character is emphasized in the species epithet, which is a compound Latin noun in the nominative case used in apposition (*inflati* is the participle in the genitive case of *inflatus* + *scapus*, the noun in the nominative singular case).

##### Type locality.

U.S.A., Utah, Sevier County, Cove Mountain, 13.4 miles south of Glenwood Fish Hatchery on FSR 068. GPS: 38.649°N, 111.950°W; elevation 9350’ (2850 m). Enormous, grazed sagebrush (*Artemisiatridentata*) meadow around Big Lake. Superficial nest under rock and in adjacent grass clump in open on gentle south facing slope; fine silty sand. Small colony (~ 400 ants). No host queens. Brood, mostly eggs and larvae, plus a few parasite queen pupae. Collected by SPC (SPC 7816), 20 July 2008.

##### Type material.

***Holotype*** queen (SPC 7816, MCZENT 00806458). ***Paratype*** male (SPC 7816, MCZENT 00806458), and the following paratypes: 63 queens, 4 males [20-VII-2008, SPC 7816]. Holotype and paratypes deposited in the MCZC. Additional paratypes deposited at CASC, CRC, LACM, and UCDC.

##### Additional material.

(i) U.S.A., Colorado, El Paso County, Black Forest, 4.5 miles south of Hodgen Rd. on Meridian Rd. GPS (from Google Maps): 39.01°N, 104.61°W; elevation 7350’ (2240 m). Open Ponderosa Pine (*Pinusponderosa*) forest, 20–40’ (6–12 m) tall with grassy understory and bearberry (*Arctostaphylos* sp.) in spots; medium sandy soil. Both colonies were under pine branches half buried in soil and litter. Collected by SPC (SPC 4117, 4120), 16 July 1994.

(ii) U.S.A., Colorado, Montrose County, 2.1 miles southeast of junction with Rt. 50 on P77 Road. GPS: 38.420°N, 107.628°W; elevation 8200’ (2500 m). Rich, mixed shrubby northeast facing slope with some sagebrush (*Artemisiatridentata*), 3–4’ (0.9–1.2 m) tall; fine sandy soil; sparse ground cover. Small colony consisting of two parasite females, ~ 30 host workers and no brood. Collected by SPC (SPC 7388), 16 July 2006.

(iii) One other collection from the type locality: SPC (SPC 8076), collected 18 July 2009, same collection data as type series, under rock in open, ~ 1000 ants, no host brood, a few inquiline pupae only.

##### Discussion and biology.

*Tapinomainflatiscapus* is a host-queen-intolerant inquiline that parasitizes *T.sessile* colonies in mid to high elevation habitats in the mountains of Utah and Colorado. So far it has been found in sagebrush meadows (*Artemisiatridentata*), mixed shrub and sagebrush, and Ponderosa Pine (*Pinusponderosa*) woodlands. Morphologically, *T.inflatiscapus* is most similar to *T.shattucki* from Massachusetts from which it can be easily distinguished by the unique shape of the antennal scape, the presence of short, erect hairs on the dorsal body surfaces, and its comparatively robust habitus. In addition, the male palp formula is 6,4. All other *Tapinoma* inquiline males have reduced palp formulae (Table 2).

*Tapinomainflatiscapus* has been collected from two localities in Colorado and a single locality in Utah. On all occasions, *T.inflatiscapus* was found in mixed colonies with its host, *T.sessile*. In every case these colonies lacked a host queen and any host brood, suggesting that *T.inflatiscapus* either kills the host queen(s) or can only colonize queenless host colonies. Parasite workers have not been observed, so *T.inflatiscapus* appears to be a workerless inquiline. We could not observe whether *T.inflatiscapus* is mono- or polygynous, although collection SPC 7388 contained two dealate queens.

The morphology of *T.inflatiscapus* queens and males reflects some characteristics of the inquiline syndrome (Fig. 2, Table 2), but the degree of specialization is not nearly as pronounced as in *T.incognitum* and *T.pulchellum*. *Tapinomainflatiscapus* alates are smaller than those of the host and approximately the size of host workers. The number of antennal segments is not reduced, and the palp count is reduced in the queens (palp formula 5,3), but not in males. The mandibles are normal in size and dentation. Both queens and males are winged and mesosomal development is robust, thus the wings appear to be functional and dispersal by flight probable. Mating may take place outside of the nest. If so, there may be less inbreeding and much better dispersal than in inquilines where mating takes place inside the host nest and where flight is problematic or impossible.

#### ﻿ Tapinoma pulchellum

Taxon classificationAnimaliaHymenopteraFormicidae

Cover & Rabeling sp. nov.

67F6E141-DA86-5EBB-8D6C-CDE091835AAC

https://zoobank.org/E469ABF5-240D-4F47-B950-1FB131C8407E

##### Diagnosis.

**Figure 3.**
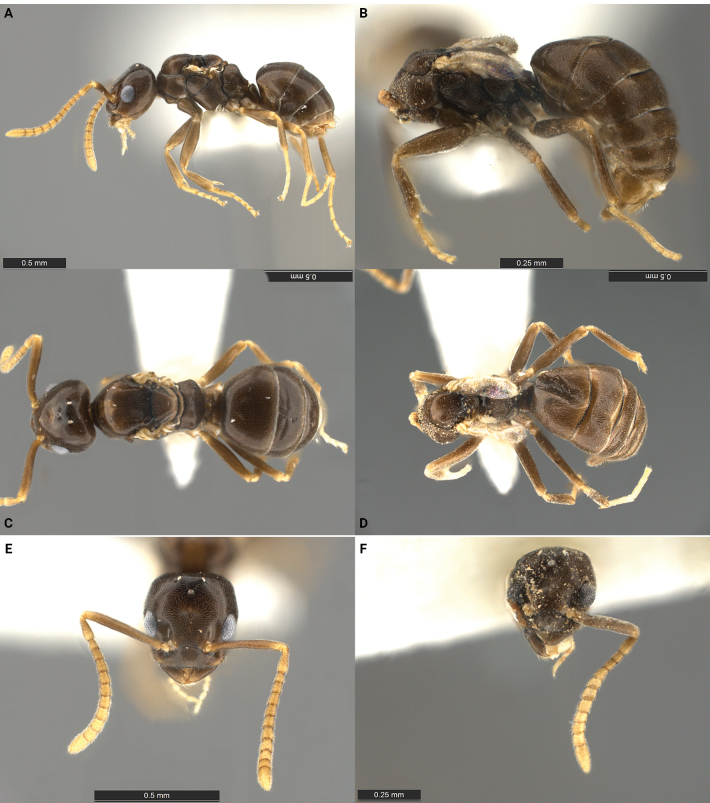
Morphological comparison of the *Tapinomapulchellum* holotype queen **A, C, E** and a paratype male **B, D, F** in lateral **A, B** dorsal **C, D** and full-face **E, F** view. The type series was collected in two adjacent pitfall traps at Eno River State Park in North Carolina. Scale bars 0.5 mm (**A, C–E**); 0.25 mm (**B, F**).

An apparently workerless, inquiline social parasite of *Tapinomasessile* exhibiting morphological traits of the inquiline syndrome. Queens and males are tiny, much smaller than the host workers, and are very similar to each other in size and habitus (Fig. 3, Table 2). Females are apparently alate, but males are brachypterous. Both sexes have a reduced (5,4) palp formula and twelve antennal segments. Females have a flat anterior clypeal border, edentate mandibles, and a petiole with a small, dorsally rounded node in side view. Females most similar to but are readily distinguished from *T.incognitum* by differing palp count, edentate mandibles, anterior clypeal border, petiole shape, and propodeal profile (subangulate with short dorsal face and long, weakly concave posterior face versus rounded convexity in *T.incognitum*).

##### Description.

***Holotype*** queen: HL 0.44, HW 0.50, SL 0.44, ML 0.76, CI 113, SI 88. Parasite queen notably smaller than host workers. Head in full-face view nearly square, dorsal margin straight with corners evenly rounded. Anterior margin of clypeus flat, median impression absent, posterior margin rounded, not projecting forward between antennal insertions. Mandibles reduced, apical tooth well developed, barely touching each other when mandibles are closed; other mandibular teeth absent. Antennae with 12 segments, scapes relatively short, surpassing the dorsal margin of head by less than their own width. Palp count 5,4. Mesosoma fully developed with typical modifications related to wing bearing. Propodeum in side view rounded, with short dorsal face and longer, slightly concave posterior face. Propodeal spiracles slightly elevated and conspicuous. Metapleural gland orifice reduced. In side view petiole with low, blunt node; viewed from the rear subrectangular in shape with dorsal margin flat to slightly convex. In dorsal view, four gastric tergites visible. Integument thin. Body surface covered with short, appressed pubescence; posterior margin of all gastric sternites and fourth gastric tergite with long erect setae. Color dark brown to yellowish brown, appendages pale yellow.

***Paratype*** male: HL 0.44, HW 0.50, SL 0.44, ML 0.76, CI 100, SI 113. Specimen damaged, but largely intact. Head glued on to point next to body. Male small, approximately the same size as the queen, brachypterous, closely similar to the conspecific queen in habitus. Head square. Eyes small, maximum diameter ~ ¼ of head length. Individual ommatidia partly fused, lacking the distinct convex surface of each ommatidium; compound eyes appear as if coated with a translucent resin. Ocelli slightly elevated above the surface the head. Anterior margin of clypeus with a broad, median, shallow impression. Mandibles reduced in size, with a single large apical tooth but lacking other teeth or denticles. Antennae with 12 segments, scapes short. Palp count 5,4. Mesosoma well-developed with typical modifications related to wing bearing. In sideview, propodeum rounded, divided into dorsal and posterior surfaces of approximately equal length. Metapleural gland orifice absent/reduced. Petiole small, overhung by first gastric tergite, not entirely visible in dorsal view. In dorsal view, five gastric tergites visible. Wings vestigial, distorted, lacking venation. Body surface densely covered with short, appressed pubescence, except for the antennal scapes and flagellum, which are covered by a dense, very short, suberect pubescence. Color medium brown, appendages yellowish brown.

##### Etymology.

*Tapinomapulchellum* is a beautiful ant (Fig. 3) and also beautifully embodies the hallmark morphological traits of the inquiline syndrome. The specific epithet *pulchellum* is the singular nominative neuter of the Latin adjective *pulchellus*, which is the diminutive of *pulcher*, meaning pretty or beautiful.

##### Type locality.

U.S.A., North Carolina, Orange County, Eno River State Park, 8 miles northwest of downtown Durham; open field adjacent to the Eno Trace trailhead. GPS: 36.073°N, 79.008°W; elevation 460’ (140 m). Large, maintained open field surrounded by mature secondary oak-hickory forest. The field was dominated by scattered *Juniperusvirginiana* to 30’ tall plus a few young *Pinusvirginiana*. Dense, grassy-herbaceous vegetation plus young Sweetgum (*Liquidambarstyraciflua*) up to 8’ tall. Sandy clay soil. The holotype queen was found in pitfall sample 13F 4,2. The paratype male was found in pitfall sample 13F 5,1. There is also a worker of the potential host from pitfall sample 13F 5,3. Collected by Amy Arnett in June 1997.

##### Type material.

***Holotype*** queen (MCZENT 00806459). ***Paratype*** male (MCZENT 00806460; same collecting locality as holotype). Holotype and paratype deposited in the MCZC.

##### Discussion and biology.

*Tapinomapulchellum* is known from only two specimens: a dealate female and a damaged male, which both exhibit typical characters of the morphological inquiline syndrome (Fig. 3, Table 2). *Tapinomapulchellum* is closely similar to *T.incognitum*, from which it can be readily distinguished by the palp formula of both queens and males, the edentate mandibles, the flat anterior clypeal border, the petiole shape, and the unique propodeal profile. This striking similarity makes it highly probable that *T.pulchellum* is a workerless inquiline, similar in its life-history to *T.incognitum*.

Both specimens were recovered from adjacent pitfall traps at the type locality in Eno River State Park in North Carolina. Accordingly, *T.pulchellum* has not been observed in mixed colonies with its host. However, *T.sessile* is the only *Tapinoma* species at the type locality, so *T.sessile* is almost certainly the host of *T.pulchellum*. Visits to the type locality in 2011 and 2012 failed to turn up additional specimens of *T.pulchellum* but allowed observations of the putative host at that site. The field contained a dense population of *Tapinomasessile*, and nests were located at the base of grass clumps or in the dense grassy thatch that covered the ground under the living vegetation. The *T.sessile* population was unusual. Colonies were large, with more than 4,000–5,000 ants, and uniformly monogynous. Both workers and queens were larger than the average size for *T.sessile*. Sexuals were not present, indicating that the mating flights had taken place already in early July.

#### ﻿ Tapinoma shattucki

Taxon classificationAnimaliaHymenopteraFormicidae

Cover & Rabeling sp. nov.

139E1346-A3B6-565C-A75D-23A65D0934C6

https://zoobank.org/573C25EE-4870-459E-82B0-C54DD83F4990

##### Diagnosis.

**Figure 4.**
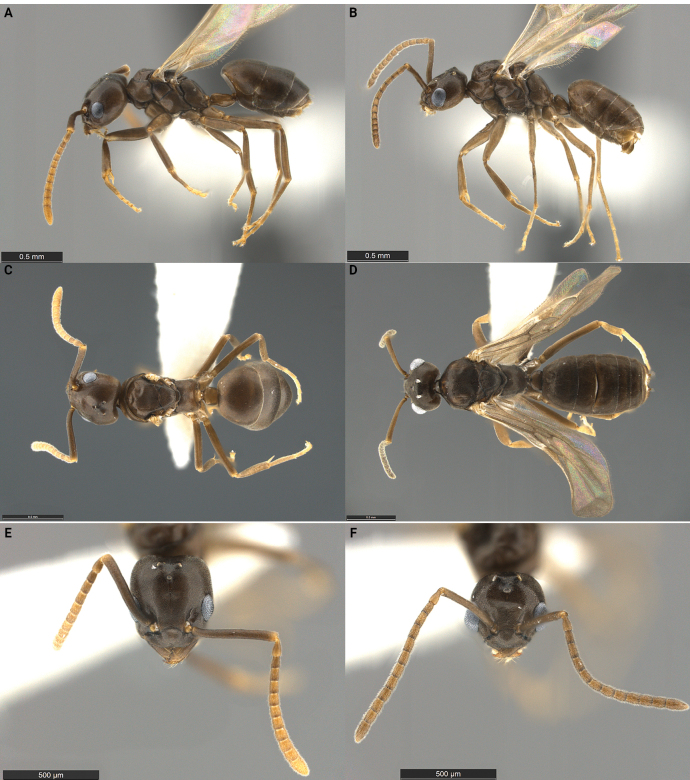
Morphological comparison of the *Tapinomashattucki* holotype queen **A, E** and a paratype male **B, D, F** in lateral **A, B** dorsal **D** and full-face **E, F** view. For the dorsal view of the *T.shattucki* queen **C**, a dealate paratype was photographed. The type series was collected in Stow, Massachusetts and belongs to a single nest series with the collection code SPC 7633. Scale bar: 0.5 mm (**A–F**).

An apparently workerless inquiline social parasite of *Tapinomasessile* showing relatively few morphological indications of its parasitic lifestyle. *Tapinomashattucki* queens and males superficially resemble those of the host, except for their smaller size, more delicate habitus, and notably reduced size of the metasoma relative to the mesosoma (Fig. 4, Table 2). Both sexes are fully alate and seem capable of flying. Female and male palp formulae reduced to 5,4. In lateral view, propodeum of female and male forming a single, flat posterior surface, and the petiolar scale is vestigial or absent. In the male, sides of head strongly convex, head nearly circular exclusive of the compound eyes in full-face view. In *T.shattucki*, males exhibit morphology similar to their free-living congeners. Females may be easily distinguished from other *Tapinoma* species by their palp count, propodeal profile, small body size, and reduced metasoma size relative to the mesosoma.

**Figure 5.**
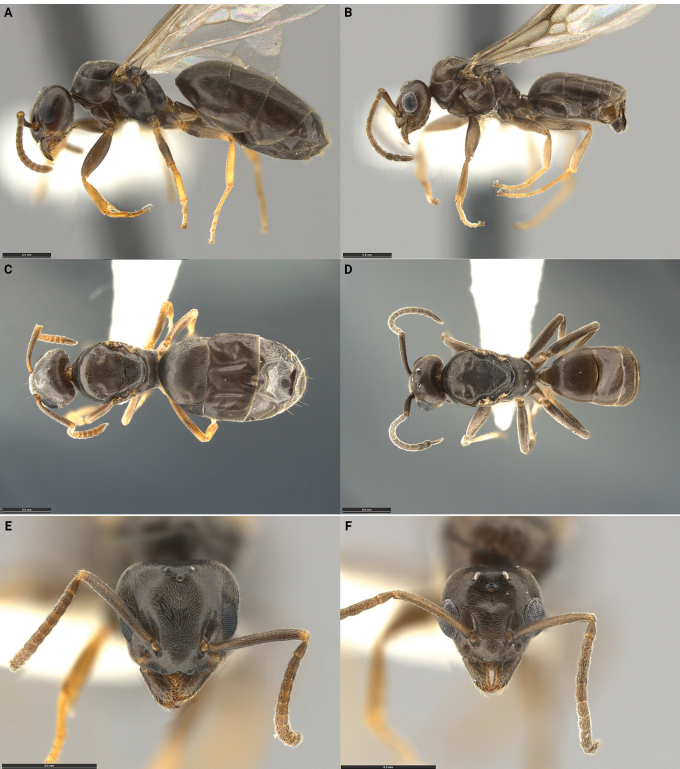
Morphological comparison of a *Tapinomasessile* queen **A, C, E** and a male **B, D, F** in lateral **A, B**, dorsal **C, D**, and full-face **E, F** view. Individuals were collected in the Black Forest in Colorado and belong to a single nest series with the collection code SPC 4118. Scale bar: 0.5 mm (**A–F**).

##### Description.

***Holotype*** queen: HL 0.65, HW 0.59, SL 0.59, ML 0.88, CI 91, SI 100. *Tapinomashattucki* superficially resembles a miniature form of *T.sessile*. Head parallel sided, slightly longer than wide (CI), dorsal margin of head straight with corners evenly rounded. Anterior margin of clypeus with broad, shallow median impression; posterior margin rounded, projecting between antennal insertions. Mandibles well developed, overlapping when closed, with 10 or 11 teeth or denticles; apical and preapical teeth well developed, distinctly larger than remaining teeth; apical tooth slightly larger than the subapical. Antennae with 12 segments, scapes long, clearly surpassing the dorsal margin of head by more than their own width. Palp formula 5,4. Mesosoma with typical modifications related to wing bearing; wings fully developed. In side view, propodeum lacking clear division into dorsal and posterior surfaces; appears as a single long, sloping posterior face. Metapleural gland orifice large and round in oblique view; orifice guarded by erect, long setae pointing inwards. Petiole small, scale absent, overhung by first gastric tergite, not visible in dorsal view. In dorsal view, three gastric tergites visible; fourth tergite mostly hidden underneath the third. Body surface covered with micro-sculpture resembling a honeycomb, and short, appressed pubescence. Color medium to dark brown, appendages yellowish to reddish brown. Paratype queens (*n* = 8): HL 0.62–0.65, HW 0.53–0.59, SL 0.59–0.62, ML 0.85–0.94, CI 86–95, SI 100–111.

***Paratype*** male: HL 0.53, HW 0.53, SL 0.50, ML 0.94, CI 100, SI 94. Medium sized males, head as wide as long (CI). Eyes large, maximum diameter little less than ½ of head width. Ommatidia clearly separated from each other; each ommatidium with a distinctly convex surface. Ocelli slightly elevated above the surface the head, but not forming a raised, triangular turret in side view. Anterior margin of clypeus straight, median impression lacking. Mandibles well developed with a single large apical tooth and ~ 14 denticles on cutting edge of mandible. Antennae with 13 segments, scapes surpassing the posterior corners of head by little less than ½ their length. Palp formula 5,4. Mesosoma with typical modifications related to wing bearing; wings well developed. In lateral view, propodeum forming a single even slope, lacking division into dorsal and posterior surfaces. Metapleural gland orifice pointing backwards, circular in posterior view. Petiole small, scale absent, overhung by first gastric tergite; not visible in dorsal view. In dorsal view, six gastric tergites visible. Integument thin, specimens shrivel when dried. Body surface densely covered with short, appressed pubescence, except for antennal flagellum, which is covered with dense, short, suberect pubescence. Color pale to medium brown, appendages yellowish brown. Paratype males (*n* = 4): HL 0.53–0.56, HW 0.53, SL 0.50–0.53, ML 0.88–0.94, CI 95–100, SI 94–100.

##### Etymology.

This species is named to honor our friend Steven O. Shattuck for his pioneering work on the ant genera of the Dolichoderinae, his invaluable contributions to the systematics of Australian ants, his important efforts in establishing the universal online ant research tool AntWiki.org, and for his and his wife Kathy’s invaluable and deeply appreciated help in reorganizing the MCZ ant collection. The species epithet *shattucki* is used as a Latin noun in the genitive case.

##### Type locality.

U.S.A., Massachusetts, Middlesex County, Stow, 47 Marlboro Road. GPS: 42.399°N, 71.524°W; elevation 230’ (70 m). Large garden adjacent to Red Maple (*Acerrubrum*) forest and reforesting wetland. Small colony in flower pot in partial shade at garden edge. Specimens preserved from 26 August to 04 September as pupae eclosed and adults matured. Collected by SPC (SPC 7633), 26 August 2007.

##### Type material.

***Holotype*** queen (SPC 7633, MCZENT 00806461). ***Paratype*** male (SPC 7633, MCZENT 00806462), and the following additional paratypes: 39 queens, 10 males [26-VIII-2007, SPC 7633]. Holotype and paratypes deposited in the MCZC. Additional paratypes deposited at CASC, CRC, LACM, and UCDC. In addition, there are 26 *T.sessile* host workers, five host males, and two host worker-queen intermorphs from the same colony, which were also deposited in the MCZC.

##### Additional material.

Wheeler’s syntype series of “*Bothriomyrmexdimmocki*” (one winged and four dealate females) comprises the only other known specimens of *T.shattucki*. The collecting locality of Wheeler’s type series is U.S.A., Massachusetts, Hampden County, Mount Tom, north of Springfield. Collected by George Dimmock, 27 August 1897. The specimens, including the lectotype worker of “*Bothriomyrmexdimmocki*” are deposited in the MCZC (MCZENT 00021289, MCZENT 00035244).

##### Discussion and biology.

This taxon has a convoluted taxonomic history. Wheeler (1915: 418) described *Bothriomyrmexdimmocki* based on “two workers, one winged, and four dealate females taken by Dr. George Dimmock August 27, 1897, from a single colony on Mt. Tom, near Springfield, Mass.” Based primarily on the unusually small size of the queens, Wheeler placed the ants in *Bothriomyrmex*, a genus of temporary social parasites that exploits *Tapinoma* hosts during colony founding, which was then thought to be exclusively Old World in distribution (Santschi 1906; but see Dubovikoff and Longino 2004; Prebus and Lubertazzi 2016). Curiously, Wheeler’s description centered on the two workers, which he was at pains to distinguish from those of the Mediterranean *Bothriomyrmexmeridionalis* (Roger). The four small, reproductive females were described afterwards and in much less detail. Emery (1925) correctly transferred *B.dimmocki* to *Tapinoma*, almost certainly without seeing the types. Creighton (1950) noted the close similarity of the *T.dimmocki* worker to that of *T.sessile*, but maintained provisional species status for *T.dimmocki*, given the problematic small size of the females. Shattuck, as part of his important re-assessment of the dolichoderine genera (Shattuck 1992), examined the types of *T.dimmocki* in the MCZC. He was able to affirm what Creighton suspected, namely the worker types of *T.dimmocki* did not belong in the genus *Bothriomyrmex*; instead, they were ordinary workers of *Tapinomasessile*. Accordingly, Shattuck (1992) synonymized *T.dimmocki* with *T.sessile* and designated one of the two worker syntypes as the lectotype of *B.dimmocki*. This was an appropriate designation, as Wheeler’s description and discussion centered primarily on the worker caste. As Shattuck remarked at the time, his taxonomic action neatly disposed of a problematic name, leaving the significance of the minute females an open question.

In late August 2007, a new collection clarified the identity of the minute females. One of us (SPC) collected a colony of *Tapinomasessile* in his garden in Stow, Massachusetts, in the bottom of a flowerpot. Surprisingly, alates of both sexes were present, though this was in late-August, a month or more later than the normal mating flights of *T.sessile* in eastern Massachusetts. In addition, the alates were notably smaller than normal *T.sessile* queens and males. A detailed examination revealed that the alates represented an inquiline parasite and that the females matched the miniature females in Wheeler’s syntype series of “*Bothriomyrmexdimmocki.*” Shattuck’s (1992) designation of a host worker as the *B.dimmocki* lectotype makes this name a junior synonym of *T.sessile*. Therefore, we describe *Tapinomashattucki* as a new species. *Tapinomashattucki* females may be readily distinguished from host queens by palp count, propodeal profile, small body size, and reduced metasoma size relative to the mesosoma.

Both times *T.shattucki* was collected, it was found in mixed colonies with its host. The type colony from Stow, Massachusetts, consisted of adult and pupal parasite females and males plus host workers and several adult host males. This suggests the possibility that, similar to *T.inflatiscapus*, *T.shattucki* might be a host-queen-intolerant inquiline that either kills the host queen(s) or preferentially exploits queenless host colonies. Two of the parasite females had swollen metasomas and were almost certainly reproductively active indicating parasite polygyny (Table 2). We did not observe any *T.shattucki* workers but instead numerous winged queens and males. Hence, our observations suggest that *T.shattucki* is a workerless inquiline social parasite of *T.sessile* and not a temporary social parasite as Wheeler (1915) suggested.

Similar to *T.inflatiscapus*, *T.shattucki* queens and males show morphological differences between sexes comparable to the sexual dimorphism observed in the host (Table 2). The morphology of *T.shattucki* queens and males shows some characteristics of the inquiline syndrome (Fig. 4, Table 2) but the degree of morphological specialization is similar to that in *T.inflatiscapus* and not nearly as pronounced as seen in *T.incognitum* and *T.pulchellum*. *Tapinomashattucki* alates are smaller than host sexuals and approximately the size of host workers. In addition, the number of maxillary palps is reduced in both queens and males (palp formula 5,4). As in *T.inflatiscapus*, the mandibles are normal in size and dentition, and both queens and males are fully winged and seem capable of flight. Hence, *T.shattucki* queens and males may mate outside the host nest and/or could disperse on the wing.

## ﻿Discussion

In this study, we describe four new species of inquiline social parasites in the dolichoderine ant genus *Tapinoma* from the Nearctic region. These social parasites represent the first inquiline species in the genus *Tapinoma* as well as the first confirmed inquilines in the ant subfamily Dolichoderinae. All four *Tapinoma* inquiline species appear to be workerless and represent at least two very different life histories (Table 2). *Tapinomaincognitum* is polygynous and the parasite queens co-exist with host queens, revealing *T.incognitum* to be host-queen-tolerant. In contrast, *T.inflatiscapus* colonies contain neither host queens nor host brood, strongly suggesting that *T.inflatiscapus* is host-queen-intolerant. *Tapinomashattucki* may also be host-queen-intolerant but we need additional collections to be certain. The life history of *T.pulchellum* is undocumented at present. However, close similarity of *T.pulchellum* to *T.incognitum* (Table 2) suggests that *T.pulchellum* might be a host-queen-tolerant inquiline as well.

For a host-queen-tolerant inquiline ant, the primary advantage of retaining the host queen(s) in the colony is the ongoing production of host workers. This secures the potential longevity of the host colony and allows the continuing reproduction of the parasite. Host queen intolerance is a more complex phenomenon. In some species, the parasite attacks and eliminates host queens some time after her acceptance by the host colony (Hölldobler and Wilson 1990; Brandt et al. 2005). Such aggressive replacement of the host queen has been observed in inquilines, such as *Leptothoraxgoesswaldi*, *Leptothoraxwilsoni*, *Monomoriumsantschii*, and *Pseudomyrmexleptosus* (Forel 1906; Kutter 1968; Klein 1987; Buschinger and Klump 1988; Heinze 1989), and also in some inquilines that likely evolved from a dulotic ancestor, such as *Temnothoraxadlerzi*, *T.birgitae*, *T.brunneus*, and *T.corsicus* (Buschinger 1989; Heinze et al. 2015).

Other so-called host-queen-intolerant inquilines do not aggressively replace the host queen but instead can only gain acceptance in host colonies that already lack a host queen (Hölldobler and Wilson 1990; Buschinger 2009). Examples include *Tetramoriumatratulum*, *Pseudoattaargentina*, and *Formicatalbotae* (Bruch 1928; Kutter 1968; Talbot 1977; Rabeling et al. 2015). Host queen intolerance is a remarkably specialized life history strategy, and in the absence of host queens, no new host workers are produced. Thus, the lifespan of the parasitized colony must be short, limited to the time that existing host workers survive, and thus limiting the parasite to one or perhaps two annual reproductive cycles. Such life histories must be sustainable only where there are large, relatively stable host populations and where there is a rapid turnover among colonies, meaning that queenless host nests occur somewhat frequently. Note that at this point, we do not know whether *T.inflatiscapus* eliminates host queens aggressively or colonizes only queenless host nests, but either way, colonies are likely comparatively short lived.

The *Tapinoma* inquiline social parasites are especially interesting because they all exploit only a single host species, *T.sessile*. Systems like this, in which a single host is exploited by multiple species of inquilines, are rare, and their evolutionary ecology is not well understood. *Tapinomasessile* is an extremely widespread and ecologically successful ant found throughout North America from central Canada to Florida and south into the Mexican highlands (Fisher and Cover 2007). *Tapinomasessile* occurs in a wide variety of habitats, nests almost anywhere, frequently moves its nest in response to changing conditions, and is often a nuisance in buildings. As one might expect, *T.sessile* exhibits variation in size, coloration, and colony structure over its enormous range. A phylogenetic analysis of *T.sessile* samples from across North America, identified four well-supported “*T.sessile*” clades (Menke et al. 2010). The analysis of DNA sequence variation in the mitochondrial marker *COI* revealed within clade genetic distances of less than 2.3% but between clade genetic distances of 7.5–10%. Accordingly, Menke and colleagues (2010) suggested that *T.sessile* might consist of a complex of cryptic species. Using genomic markers, we are revisiting these findings with a special emphasis on the evolutionary origin of the inquiline social parasites. Recent studies of inquiline social parasite evolution have revealed several convergent pathways by which inquilinism evolves. These include inquiline parasites speciating directly from their future hosts in sympatry, inquilines originating in allopatry and via host-shift speciation, as well as inquilines transitioning secondarily from a different parasitic life history, such as temporary social parasitism and dulosis, to inquilinism (e.g., Rabeling et al. 2014; Heinze et al. 2015; Messer et al. 2020; Borowiec et al. 2021; Degueldre et al. 2021; Ward and Branstetter 2022; Mera-Rodríguez et al. 2023). We are confident that further studies of host-parasite co-evolution in *Tapinoma* will reveal novel insights into the complex mosaic evolution of ant inquiline social parasites.

## Supplementary Material

XML Treatment for Tapinoma incognitum

XML Treatment for Tapinoma inflatiscapus

XML Treatment for Tapinoma pulchellum

XML Treatment for Tapinoma shattucki

## ﻿Additional information

### Author ORCIDs

Stefan P. Cover https://orcid.org/0009-0000-1911-0283

Christian Rabeling https://orcid.org/0000-0003-1292-0309

### Data availability

All of the data that support the findings of this study are available in the main text.
